# Disseminating a cervical cancer screening program through primary physicians in Hong Kong: a qualitative study

**DOI:** 10.1186/1472-6963-14-85

**Published:** 2014-02-25

**Authors:** Cecilia S Fabrizio, Christopher M Shea

**Affiliations:** 1 School of Public Health, University of Hong Kong, 5th Floor, William MW Mong Block, 21 Sassoon Road, Pokfulam, Hong Kong, SAR, China; 2 Department of Health Policy and Management, Gillings School of Global Public Health, University of North Carolina-Chapel Hill, Chapel Hill, NC, USA

**Keywords:** Cervical cancer screening, Dissemination, Physicians, Prevention, Screening programs

## Abstract

**Background:**

Organized screening programs are more effective and equitable than opportunistic screening, yet governments face challenges to implement evidence-based programs. The objective of this study was to identify reasons for low levels of adoption among primary care physicians of a government sponsored Cervical Screening Program (CSP).

**Methods:**

We conducted in-depth interviews with a snowball sample of primary care private and public primary care physicians in Hong Kong. Rogers’ theory of diffusion of innovation was used to understand the factors that influenced the physicians’ practice decisions.

**Results:**

Our study found that Hong Kong physicians made the decision to encourage cervical screening and to participate in the CSP based primarily upon their clinical and business practice needs rather than upon the scientific evidence. The low rates of adoption of the CSP can be attributed to the physicians’ perceptions that the program’s complexity and incompatibility exceeded its relative advantages. Furthermore, women’s knowledge, attitudes and practices, identified as barriers by physicians, were also barriers to physicians adopting the CSP.

**Conclusions:**

In both private and public health care systems, screening programs that rely on physicians must align program incentives with the physicians’ motivators or pursue additional demand creation policies to achieve objectives.

## Background

Cervical cancer is relatively unique among cancers in that its cause is known, it can be identified early and it can be treated effectively at a pre-cancerous stage. Cervical screening is most efficiently conducted as part of an organised screening program; that is a program organised at the national or regional level, with explicit evidence-based policies that include target populations, frequency and treatment [1]. Compared to opportunistic screening, which is done at the request of an individual or her physician, organised programs have greater impact on cancer incidence and mortality, more potential to reduce disparities, higher cost effectiveness, and larger effects on risk reduction for a population [2].

Hong Kong women suffer from a disproportionately higher incidence of preventable morbidity and mortality from cervical cancer than women in similarly industrialized countries [3]. Hong Kong’s poorer results are attributed to the low rate of screening among women overall, and to low screening rates among older and low-income women in particular [4]. The major barriers to screening among women in Hong Kong include age, lack of knowledge about the need for screening, socio-economic position, and marital status, and are similar to the barriers identified globally [5-10]. Research also indicated that the women most at risk were more likely to report that their doctor did not order or recommend the screen, or that they did not know they needed a screen. Price has not been identified as a major barrier.

Hong Kong has a public-private health care system, with the majority of outpatient primary care visits (about 85%) attributed to private medical or alternative medical practitioners [11]. In the primary care system, physicians with no specialty training are described as in General Practice, while those in Family Medicine have received fellowship certification in Family Medicine. In this mixed public-private system, cervical screening in Hong Kong has been conducted opportunistically by private practitioners or as part of well-women health checks. This screening was inefficient and inequitable, with poor coverage of those at risk, and frequent screening of a minority of women that were more likely to be younger and have more education [4]. In 1998, this opportunistic system was estimated to be equivalent to an organised program with only 50% coverage and 10-year screening intervals [12].

In 2004, the Hong Kong government introduced the Cervical Screening Program (CSP), an organised program with the objectives of increasing the population screening coverage rate and reducing the incidence and mortality of cervical cancer [3]. Both physicians and women were eligible to join individually. The CSP’s evidence-based program components included recommended age and frequency targets, quality assurance, capacity for follow-up and treatment, and a registry for surveillance and analysis. However, the CSP lacked other evidence-based components, such as a national call-recall system, interventions to increase provider screening recommendations, targeted interventions toward those most at risk, and efforts to reduce inappropriate over-screening. The CSP also did not provide any subsidies to women to access care, nor to providers to increase participation. Eight years after its introduction, registration in the CSP is below 20% among both physicians and women [13]. Population-based tracking data reported women’s ever-screened rate of 63.3% in 2010, essentially unchanged since the program began. In addition, overall screening efficiency declined, with the rate of over-screened, low-risk women (those who report screens “once or more a year”) up from 56.0% in 2003, pre-initiation of the CSP, to 62.8% in 2010 [3,14]. These results indicate that the current structure and/or dissemination strategy of the CSP may need to be modified.

The purpose of this study was to analyze physicians’ attitudes toward the CSP to identify reasons for low levels of adoption of this organised cervical cancer screening program. We drew upon Rogers’ Diffusion of Innovation (DOI) theory [15] to guide the study. Rogers defines adoption as “full use of an innovation as the best course of action available” and rejection as a decision “not to adopt an innovation” [15], p. 177. Furthermore, he proposes five attributes or characteristics of innovation that influence the adoption process: relative advantage, compatibility, complexity, trialability, and observability. Our research questions drew upon these characteristics to explore the adoption decision: 1) What are the physicians’ perceptions of the benefits of the CSP? 2) What are the physicians’ perceptions of the program’s complexity? 3) How compatible is the CSP with the physicians’ established practices? 4) What is the perceived trialability of the CSP? and 5) How observable is a physician’s participation in the CSP to patients and other providers? We believe that this work offers lessons for the international public health community because it highlights barriers to dissemination of public health programs that are common to both private and public primary health care systems.

## Methods

We used the Hong Kong College of Community Medicine as the sampling frame to identify Hong Kong physicians whose practice included women in the screening target age. Physicians were included if they were general practice physicians or obstetrician/gynecological specialists, and excluded if they did not speak English at a level needed to conduct the interview. The sampling strategy utilized both snowball sampling, after the initial referrals from the physicians’ association leaders and HKU School of Public Health professors; and purposeful sampling, to include a representative mix of genders, specialties, districts and CSP-registered physicians. Sampling was more difficult than anticipated, as only about half of the physicians interviewed were willing or able to recommend a colleague to interview. In an effort to recruit additional physicians in different districts, particularly more men, two physicians were recruited by “cold calling” physicians on an insurance directory listing. Furthermore, three physician leaders, two of which were known professionally to the lead author, were interviewed to explore their perceptions of their association members, as well as their own experiences with the CSP. Finally, due to a lack of private practitioners who were knowledgeable about the program, the sample also included three physicians who worked in government or government-sponsored NGO clinics (“clinic-based physicians”), which required their physicians to register with the program.

All interviews were conducted in English, the language of medical education in Hong Kong and there were no language issues that affected acquisition or interpretation of the interview data. Data collection continued until data saturation was reached pertinent to the three themes identified. The interviews were conducted over the period of January to March 2011. Written consent was obtained from each participant. The Institutional Review Board of the University of Hong Kong and the University of North Carolina at Chapel Hill, Office of Human Research Ethics approved the study.

The interviews focused on the physicians’ perception of the characteristics of the program and the elements that influenced their adoption decision. Given that relatively few physicians were familiar with the CSP, the interviews also included a discussion about a similar government program, a flu vaccination subsidy. This voluntary program offered older residents a voucher to offset the cost of the vaccine given in a private physician’s office. Similar to the structure of the CSP, this program was a public-private partnership, aimed at a universal population, and with many administrative requirements.

### Data analysis

Coding and analysis were conducted using Dedoose (Dedoose.com). Rogers’ theoretical framework guided development of the codes, a priori. The first author coded each interview transcript and created code summaries, which were discussed with the second author. To increase study reliability, all of the interviews were double-taped, the transcripts were re-checked against the audiotape and post-interview contact summary notes as they were coded. Also, check-coding (i.e., recoding transcripts to check for consistency) was used periodically to assess for potential code drift [16]. In order to improve validity, interview summaries were examined for discrepant viewpoints. Quotes were used to illustrate themes and to present discrepant findings.

## Results

A total of 24 physicians were identified and 16 physicians accepted, for a response rate of 67%. Six physicians (38%) were registered with the CSP and 12 (75%) were women (Table 1). Seven (44%) of the respondents were in Family Practice; seven (44%) were in General Practice (GP); and two (13%) were OB/GYNs; doctor. Five physicians were in solo practice (31%); five practiced in small groups of six or less (38%); and the remaining five (31%) worked for government or large NGO multi-site practices. The respondents had a range of experience, from six to 25 years, and the sample was geographically and economically heterogeneous.

**Table 1:** Characteristics of physician sample

**Characteristics**		**Number (n = 16)**	**Percentage**
Gender			
	Female	12	75%
	Male	4	25%
Specialty			
	General Practice	7	43.8%
	Family Practice	7	43.8%
	OB/GYN	2	12.5%
Years in practice			
	≤ 10	3	18.8%
	11 ≤ 20	5	31.3%
	> 20	8	50.0%
Practice size			
	One physician	5	31.3%
	Small office (2–6 physicians)	6	37.5%
	Large clinic/multi-site	5	31.3%
Average cervical screens per month			
	≤10	7	43.8%
	11 ≤ 50	3	18.8%
	> 50	6	37.5%
Registered with CSP program			
	Yes	6	37.5%
	No	10	62.6%

Three themes, each pertinent to Rogers DOI theory, emerged from the physician interviews (Table 2).

**Table 2:** Frequency of code usage

**Code name**	**Times code used**	**Code definition **[15]	**Illustrative quotation**
Relative advantage (benefit)	67	Degree to which an innovation is perceived as better than the idea it supersedes.	“You either determine to do this kind of screening and you would have your own system to make sure this follow-up or basically this is not something high priority in your practice”.
Complexity	26	Degree to which an innovation is perceived as difficult to understand and use.	“After you try it a few times you find it’s difficult; it’s just not worthwhile.”
Compatibility	22	Degree to which an innovation is perceived as being consistent with the existing values, past experiences, and needs of potential adopters.	“It’s odd if you go see a doctor and then you mention you thought of cervical screening. Will she think I’m trying to get more money from her?
Trialability	5	Degree to which an innovation may be experimented with on a limited basis.	“Actually I signed up but never used it.”
Observability	8	Degree to which the results of an innovation are visible to others.	“If someone was already using the system and they are very excited about it and they are telling me how the system has been able to help their patient care.”

### Theme 1: The CSP’s benefits were rarely meaningful to the physicians’ practices

Most physicians identified the capability for recalling their registered patients who had previously been screened, and accessing those registered patients’ online screening history as the most notable benefits of the CSP. However, these benefits were not substantially better than their current practices. For example, nine of the 10 physicians that performed even a minimal number of Pap tests (at least 10 screens a month) already had a recall system. Most physicians felt that a recall system should be an essential component of any general practice, for business reasons:

*If the physicians don’t recall the women, the women won’t come back. The physicians will lose the business, right? That is why in that sense the Registry is of limited help to one’s practice. You either determine to do this kind of screening and you have your own system to make sure this follow-up happens, or basically this is not something that is a high priority in your practice *(Family Practice physician; registered with the CSP).

Although most physicians preferred to have access to their patient’s past screening results to improve patient care, many physicians perceived the online system to be unnecessary, as physicians kept their regular patients’ past records in their files and new patients generally knew a sufficient amount about their past screening results for the physicians to determine their clinical history.

*Pap smear results are simple. The patient will know if her past result is normal negative, or if there is something abnormal that requires some follow-up afterward….So the information is already adequate* (OB/GYN physician; not registered with the CSP).

Five of the six physicians registered for the CSP did not perceive any benefits from the program.

For a point of comparison against which to assess perceived value of the CSP, participants were asked about the value of the flu voucher program. This flu voucher, which was a subsidy for patients that could be used with any private provider, often was passed on as physician revenue during an incremental visit. The physicians preferred this program to the CSP because of the increased patient demand and the subsidy.

*Why did I participate (in the flu vaccine program)? I have lots of patients enrolled. So I think that it’s providing an extra service to my patients…a lot of patients…a lot more than from the cervical screening [program]* (General Practice physician; registered with the CSP).

*Why did I join the flu scheme instead of the CSP? Because I get revenue from it *(Family Practice physician; not registered with the CSP).

In summary, the study physicians felt that the CSP did not fulfill an information need or have a positive financial impact, and that it was not demanded by their patients.

### Theme 2: CSP procedures were perceived as costly and complex

Among the respondents who were familiar with the CSP, the program was almost universally perceived to be problematically complex. Often the physicians reported that the program’s complexities negatively impacted their practice costs or revenues. Physicians complained about the staff training time required and the program’s on-going administrative issues:

*Well, it [administration] is complicated. Trying to explain to the patients what to sign; filling in all the forms; sending them in. If you are late you don’t get paid. If the patient signs the wrong thing, you don’t get paid. And you end up losing money. The patient never comes back to pay *(General Practitioner physician; not registered with the CSP).

The flu vaccine was also perceived as administratively complex. One physician succinctly summarized both programs’ onerous administrative burdens: “Similar. Just as bad.”

Physicians placed differing levels of importance on the CSP’s complexity as a barrier to enrollment depending on the degree to which their administrative staff could manage the work. For those few physicians who had the administrative capability, this complexity was manageable; however, most physicians had limited staff capability. In Hong Kong, since few patients have insurance coverage that requires physicians to complete burdensome paperwork, physicians typically have minimal staff for non-clinical care.

### Theme 3: many physicians found the CSP policies inconsistent with their usual clinical and business practices

All the physicians interviewed stated their belief that cervical screening was perhaps *the* most important preventive care service, and most of the physicians interviewed said that they often raised the issue with their patients. However, among physicians that regularly conducted cervical screens, many raised concerns about the CSP’s compatibility with their preferred patient care practices for clinical and business reasons. Most physicians stated that the CSP’s recommended three-year frequency was incompatible with their usual practice, as they needed to be flexible, for either clinical (patient) or customer satisfaction (business) reasons. Sometimes physicians used annual screens to trigger more complete check-ups:

*Some of them [patients] are on the pill and they’re actually having more partners than they like to admit. So really part of the Pap smear screening isn’t just a Pap smear screening; it’s also sexually transmitted disease screening. So in fact I am merging the two things together but that is working within the context of being a doctor. If you look at just cervical screening on its own, then there is nothing wrong with it [screening every three years]. But of course the family physician like me is looking at the patient as a holistic person *(Family Practice physician; not registered with the CSP).

Other times the patients requested annual screens and the physicians felt obligated to conduct one or she/he risked losing the patient to a more accommodating physician:

*A lot of the Pap smears I do are actually people who suggested that they wanted a health check, so they do it every one to two years anyway. So they are the people that are probably the ‘worried well’. So, yes, they actually have a screen every year, so if I follow the CSP [frequency policy] I will lose my client. It will actually lose business for me *(Family Practice physician; not registered with the CSP).

Because some physicians screened as many as 50-100 women per month, they reported that reducing the frequency of their patients’ cervical screens from every year to every three years would likely reduce their revenue from these preventive care visits significantly, by as much as two-thirds.

Among those physicians that rarely screened their patients, many had attempted to increase screening rates, but they faced barriers due to women’s knowledge, attitudes and behaviors (KAP). For physicians serving women in the lower socio-economic strata, their patient population had little knowledge of cervical screening and most physicians were reluctant to spend time educating and motivating their patients.

*We have a lot more women with whom you would have to reach out to initiate screening, because they don’t have very much knowledge about it. With the local population, if they don’t feel that they have anything wrong with them, then they don’t feel the need to do screening. They really don’t. The whole concept of this kind of preventive screening is not very well established in the local Chinese population. They are very inactive rather than proactive* (General Practice physician; not registered with the CSP).

At least half of the physicians who rarely screened tended to have a fluid patient base as Hong Kong people often change physicians frequently, even during the same episode of illness. These physicians expressed concern that new patients would be suspicious of opportunistic recommendations and they spoke about needing to build trust before recommending screening to their newer patients:

*It [opportunistic screening] is one of the routine questions I ask, especially with new patients. I usually wouldn’t tell them to do it. I mean I’ll tell them, I’ll ask them, but they usually won’t do it right away. The reason being that I don’t want to make it sound like I want to do it…because it’s not free for them. They have to pay for it. I want to increase their readiness, but I don’t want them to think that I am trying to do business in a way *(General Practice physician; not registered with the CSP).

Even among women likely to undergo screening, physicians faced barriers, including women’s preferences for female screeners or gynecological specialists. One male physician who had trained overseas had restricted himself to simply reminding women to be screened, rather than suggest that he perform the screen himself:

*Some patients prefer female practitioners. They are just not comfortable with a male doctor. So for these patients I would not insist. But I will always remind them to make sure they do have a gynecological checkup with a Pap smear every two years *(Male, Family Practice physician, registered with the CSP).

*Some people ask me, “I want to have a cervical screen. Can you introduce a specialist gynecologist for me?” I said: “Have you talked with the doctor that you usually go to see?” [And they reply] “No, I am not going to talk to him. I think it has to be done by a gynecologist specialist for this” *(Female, Family Practice physician, not registered with the CSP).

These physicians found it almost futile to make the effort to encourage their patients to be screened and therefore did not see the benefit to enrolling in the CSP.

## Discussion

Our study found that Hong Kong physicians made the decision to encourage cervical screening and to participate in the CSP or to follow its guidelines based primarily upon their clinical and business practice needs rather than upon the scientific evidence. The low rates of adoption of the CSP can be attributed to the physicians’ perceptions that the program’s complexity and incompatibility exceeded its relative advantages. Furthermore, women’s knowledge, attitudes and practices, identified as barriers by physicians, were also barriers to physicians adopting the CSP. Our study finding of the physicians’ negative perceptions of the program, coupled with the individual nature of the patient–physician screening decision, indicate that the program’s strategic focus on the private sector physician should be re-assessed to either add physician incentives or to refocus on increasing population-based demand.

Both private and public health care systems have struggled to disseminate evidence-based screening programs among primary care physicians; however, although Rogers DOI theory has been applied in such diverse fields as international development, education and HIV [17], there is little evidence on the application of Rogers’ DOI on cancer screening guidelines. Glasgow et al.’s report [18] on a National Cancer Institute-sponsored workshop acknowledged the need to design dissemination into the planning stage of a policy and recommended that theories or models should guide implementation.

In England, physician adoption of a cervical screening program in the 1990’s was not broadly successful until motivators and incentives were aligned. Initially the program did not offer a financial incentive, but when it was added screening coverage rose from 42% to 85% from 1990 to 1998, with over 90% of physicians reaching a target of over 80% of their patients’ screened [19]. In the United States, although policy makers typically worked with the private sector to issue screening guidelines, surveillance of primary care physicians’ cervical screening practices shows that many physicians did not follow screening guidelines during the period 2005-2010, for similar clinical and business practice reasons to those shown in this study [20]. Furthermore, a 2011 survey of obstetrician-gynecologists reported that only approximately half of these specialists followed guidelines for Pap and HPV testing [21]. Demand-generating policies, such as the CDC’s National Breast and Cervical Cancer Early Detection Program, have had some success in increasing targeted breast and cervical screening coverage by providing subsidies to the most underscreened [22].

Consistent with these experiences in the U.S. and England, our study suggests two important lessons for public health programs. First, when a program depends upon physicians to achieve its goals, the program incentives must align with physicians’ motivations. In a private primary care system, the financial viability of their primary care practice is a key motivator, so financial incentives may be warranted. Also notable, however, is the lack of active participation among the physicians employed by semi-public clinics because their institutions managed most of the administrative aspects of the program. Therefore, even with the potential to mitigate the complexity of the program (Theme 2), and although business priorities did not apply (Theme 3), the lack of perceived benefits (Theme 1) impeded these physicians’ participation in the program. Second, governments may be more successful by focusing on demand-generating policies that they can control, such as population-based invitations, subsidies for high-risk women, and expanded capacity in larger clinic-based settings. These will encourage private physicians to overcome their objections to the administrative requirements.

This study had some limitations. First, it was difficult to identify and recruit private physicians who were both registered and active in the program, so the findings may not adequately represent the perspectives of all private physicians. However, perceptions of the registered private physicians interviewed were consistent with those of the unregistered physicians, and with the semi-public clinic-based registered physicians. Second, because our study was qualitative and exploratory, the results are not generalizable to other settings and programs. However, we believe the study could inform hypotheses for future studies focusing on the CSP participation rates, or adoption decisions of other programs. Third, although 70% of Hong Kong physicians were estimated to be male [23] only four of the 16 respondents (25%) were men, due to a disproportionate number of female physicians referred during the recruitment process, as well as a substantial difference between response rates for female versus male physicians. However, given women’s screening preference for female physicians, this group may have been more likely to have been involved in the CSP and more knowledgeable about cervical screening practices. Finally, prior to the study, the primary author already knew two of the physicians interviewed. This professional relationship could have introduced bias. However, we do not believe such bias was an issue in our study because interviews with these physicians’ yielded data that was consistent with interviews of physicians who were not known to the researcher.

## Conclusions

Organised screening programs are more effective and equitable than opportunistic screening yet governments are challenged to implement evidence-based programs through primary care physicians. This study suggests that the individual clinical and business needs of private physicians can impede public health program dissemination. Even physicians in semi-public settings, without the same business needs of their private practice counterparts, are unlikely to embrace a program if the benefits are not perceived as valuable. Therefore, programs need to ensure benefits are observable to physicians and incentives are aligned with their motivations. If a program is unable to do so, it might be best served by pursuing demand-creation strategies that are not reliant upon the physicians.

## Competing interests

The authors declare they have no competing interests.

## Authors’ contributions

CF was the principal investigator of the study and took the lead role in conceptualizing and drafting the manuscript. CS contributed to the conceptualization and editing of the manuscript. Both authors read and approved the final manuscript.

## Pre-publication history

The pre-publication history for this paper can be accessed here:

http://www.biomedcentral.com/1472-6963/14/85/prepub
